# Hemoglobin A1C as a prognostic factor and the pre-diabetic paradox in patients admitted to a tertiary care medical center intensive cardiac care unit: The Jerusalem platelets thrombosis and intervention in cardiology (JUPITER-6) study group

**DOI:** 10.1186/s12933-022-01529-1

**Published:** 2022-05-30

**Authors:** Lior Lupu, Louay Taha, Rivka Farkash, Feras Bayya, Mohammad Karmi, Yoed Steinmetz, Fauzi Fadi Shaheen, Nimrod Perel, Kamal Hamayel, Nir Levi, Tommer Maller, Hani Karameh, Gavriel Lichewitz, Dov Gavish, Nurit Algur, Michael Glikson, Elad Asher

**Affiliations:** 1 Department of Cardiology, Tel Aviv Medical Center (Affiliated to The Sackler Faculty of Medicine, Tel Aviv University, Tel Aviv, Israel), Weizmann 6, 6423919 Tel Aviv, Israel; 2 Department of Cardiology, Jesselson Integrated Heart Center, Shaare Zedek Medical Center (Affiliated to The Faculty of Medicine, Hebrew University of Jerusalem, Jerusalem, Israel), Jerusalem, Israel

**Keywords:** Diabetes mellitus, Prediabetes, Hemoglobin A1c (HbA1c), Acute cardiac care, Prognosis

## Abstract

**Background:**

Hemoglobin A1C (HbA1c) is a form of glycated hemoglobin used to estimate glycemic control in diabetic patients. Data regarding the prognostic significance of HbA1c levels in contemporary intensive cardiac care unit (ICCU) patients is limited.

**Methods:**

All patients admitted to the ICCU at a tertiary care medical center between January 1, 2020, and June 30, 2021, with documented admission HbA1c levels were included in the study. Patients were divided into 3 groups according to their HbA1c levels: < 5.7 g% [no diabetes mellitus (DM)], 5.7–6.4 g% (pre-DM), ≥ 6.5 g% (DM).

**Results:**

A total of 1412 patients were included. Of them, 974 (69%) were male with a mean age of 67(± 15.7) years old. HbA1c level < 5.7 g% was found in 550 (39%) patients, 5.7–6.4 g% in 458 (32.4%) patients and ≥ 6.5 g% in 404 (28.6%) patients. Among patients who did not know they had DM, 81 (9.3%) patients had high HbA1c levels (≥ 6.5 g%) on admission. The crude mortality rate at follow-up (up to 1.5 years) was almost twice as high among patients with pre-DM and DM than in patients with no DM (10.6% vs. 5.4%, respectively, p = 0.01). Interestingly, although not statistically significant, the trend was that pre-DM patients had the strongest association with mortality rate [HR 1.83, (95% CI 0.936–3.588); p = 0.077].

**Conclusions:**

Although an HbA1c level of ≥ 5.7 g% (pre-DM & DM) is associated with a worse prognosis in patients admitted to ICCU, pre-DM patients, paradoxically, have the highest risk for short and long-term mortality rates.

## Background

Type 2 diabetes mellitus (DM) is a known risk factor for cardiovascular diseases [1], and patients with DM and cardiovascular disease suffer from higher morbidity and mortality as compared with non-diabetic patients [2–4]. Moreover, studies have shown a progressive relationship between plasma glucose levels and cardiovascular risk, and even pre-DM patients are at increased risk for cardiovascular diseases [5]. Also, in patients hospitalized due to acute coronary syndrome (ACS), a higher plasma glucose level at admission is associated with higher mortality risk. This association is seen both in patients with and without a diagnosis of DM [6, 7].

Glycated hemoglobin A1c (HbA1c), the major fraction of glycated hemoglobin, is formed by irreversible non-enzymatic glycation. Its concentration depends only on the red blood cell life span and blood glucose level [8]. Thus, it is an indicator for blood glucose concentrations in the preceding 2–3 months. It is of great significance for monitoring the regulation of diabetes and the risk for complications. Furthermore, the American Diabetes Association (ADA) and World Health Organization (WHO) recommend using HbA1c for the diagnosis of DM [9, 10].

Data regarding HbA1c and outcomes in contemporary intensive coronary care units (ICCUs) is limited. An observational study performed in a medical intensive care unit (MICU) found that HbA1c testing in patients with stress hyperglycemia during hospitalization reveals undiagnosed diabetes in 14% of patients. Moreover, hyperglycemia with lower baseline HbA1c was associated with increased mortality as well [11]. Furthermore, in patients with ACS, acute glycemic control, as estimated by plasma glucose levels, rather than the chronic pre-existing glycemic state as estimated by HbA1c affects prognosis [12, 13]. Hence, we aimed to investigate the prognostic significance of admission HbA1c levels among contemporary ICCU patients in a tertiary care medical center.

## Methods

### Study population

We performed a prospective single-center observational cohort study at the Shaare Zedek Medical Center, a tertiary referral hospital and one of the 2 largest medical centers in Jerusalem. The study population consisted of non-selected consecutive patients admitted to the ICCU between 1 January 2020 and 30 June 2021. We included only patients for whom HbA1c levels on admission were documented. Patients were divided into 3 groups according to their HbA1c levels: < 5.7 g% [no diabetes mellitus (no-DM)], 5.7–6.4 g% (pre-DM) and ≥ 6.5 g% (DM). The division into groups was done according to the position paper of the ADA: Classification and Diagnosis of Diabetes: Standards of Medical Care in Diabetes—2019 [10]. Obesity was defined by a body mass index (BMI) > 30. Demographic data, comorbid conditions, medications, physical examination, laboratory findings, in-hospital complications, length of stay (LOS), and in-hospital mortality were systematically recorded. In-hospital complications were defined as the occurrence of acute heart failure, left ventricular thrombus, shock, recurrent myocardial infarction or stent thrombosis, malignant arrhythmias, mechanical complication (free wall rupture or ventricular septal rupture), acute renal failure, severe bleeding requiring blood transfusion, vascular complication, cerebrovascular accident, anoxic brain damage and sepsis.

### Study outcomes

The primary outcome of our study was overall mortality, with a follow-up of up to 1.5-year from the time of index hospitalization. Every death in Israel is documented in a central database of the Israeli Ministry of the Internal Affairs and is updated in the hospitals’ medical records. We used these records to examine the overall mortality rates among the study participants. The study’s secondary outcomes included: (a) comparison of patients’ characteristics among the different HbA1c levels; and (b) in-hospital interventions and complications during the index hospitalization among the different HbA1c levels.

### Statistical methods

Patients’ characteristics were presented as numbers (%) for categorical variables and as means (SD) or medians (IQR) for normal and non-normal distributed continuous variables, respectively.

A chi-square test was used for the comparison of categorical variables. Analysis of variance (ANOVA) test and Kruskal–Wallis test were performed for comparison of normally and non-normally distributed continuous variables, respectively. For the post-hoc analyses, we used the Bonferroni correction method.

Hazard ratios (HRs) and corresponding 95% confidence intervals (CIs) for the association between the HbA1c group and mortality were estimated using a Cox proportional-hazards model. The model included the following potential confounders: age, gender, hypertension, hyperlipidemia, diabetes mellitus diagnosis, in-hospital complication, prior intervention, heart failure, and chronic kidney disease. Data were censored at death or at the end of the study period. Kaplan Meier survival curves were compared using the log-rank test. Then, the pre-DM and DM were grouped together and the same analyses were performed.

All tests were conducted with a two-sided overall 5% significance level (α = 0.05). All analyses were performed using R software (R Development Core Team, version 4.0.3, Vienna, Austria).

### Results

A total of 1739 patients were included in the study. HbA1c on admission was documented in 1412 (81%) patients. Of them, 550 (39%) patients were defined as no-DM, 458 (32.4%) were defined as pre-DM and 404 (28.6%) were defined as DM patients (Table 1).

**Table 1:** Demographic and baseline characteristics of patients stratified by HbA1c

	Groups			p-value
	HbA1c < 5.7 g% (No-DM)	5.7 g% ≤ HbA1c < 6.4 g% (Pre-DM)	HbA1c ≥ 6.5 g% (DM)	
n = 1412	550 (39%)	458 (32.4%)	404 (28.6%)	
Age (years)				< 0.001
< 40	32 (9.7%)	3 (1.2%)	4 (1.9%)	
40–49	36 (10.9%)	6 (2.4%)	9 (4.2%)	
50–59	54 (16.3%)	43 (17.1%)	37 (17.5%)	
60–69	60 (18.1%)	63 (25.0%)	63 (29.7%)	
70–79	74 (22.4%)	66 (26.2%)	63 (29.7%)	
80–89	60 (18.1%)	60 (23.8%)	30 (14.2%)	
> 90	15 (4.5%)	11 (4.4%)	6 (2.8%)	
Gender (male)	368 (66.9%)	307 (67.0%)	299 (74.0%)	0.035
Obesity (BMI > 30)	27.0 [23.5, 30.0]	28.26 [24.4, 31.2]	28.9 [25.5, 31.6]	< 0.001
Hypertension	262 (47.6%)	297 (64.8%)	315 (78.0%)	< 0.001
Hyperlipidemia	216 (39.3%)	253 (55.2%)	282 (69.8%)	< 0.001
A diabetes mellitus history	61 (11.1%)	158 (34.5%)	323 (80.0%)	< 0.001
Tobacco use	162 (29.5%)	115 (25.1%)	123 (30.4%)	0.168
Ischemic heart disease	125 (22.7%)	133 (29.0%)	172 (42.6%)	< 0.001
Family history of coronary artery disease	44 (8.0%)	27 (5.9%)	21 (5.2%)	0.180
Cerebrovascular disease	37 (6.7%)	23 (5.0%)	42 (10.4%)	0.080
Peripheral artery disease	18 (3.3%)	19 (4.1%)	36 (8.9%)	< 0.001
Chronic kidney disease	62 (11.3%)	68 (14.8%)	70 (17.3%)	0.026
Chronic heart failure/cardiomyopathy	65 (11.8%)	69 (15.1%)	79 (19.6%)	0.04
Chronic obstructive pulmonary disease/asthma	39 (7.1%)	42 (9.2%)	29 (7.2%)	0.407
Pulmonary hypertension	14 (2.5%)	27 (5.9%)	24 (5.9%)	0.013
Cognitive decline	18 (3.3%)	8 (1.7%)	13 (3.2%)	0.272
Malignancy	49 (8.9%)	45 (9.8%)	30 (7.4%)	0.458
Anemia	32 (5.8%)	15 (3.3%)	27 (6.7%)	0.060
Atrial fibrillation	53 (9.6%)	66 (14.4%)	55 (13.6%)	0.05

Data are reported as n (%), or median (25th–75th quartile)

DM, diabetes mellitus; BMI, body mass index

Interestingly, 81/870 (9.3%) patients had high HbA1c levels ≥ 6.5 g% on admission, but did not know they had DM.

### Patients characteristics

**Fig. 1:**
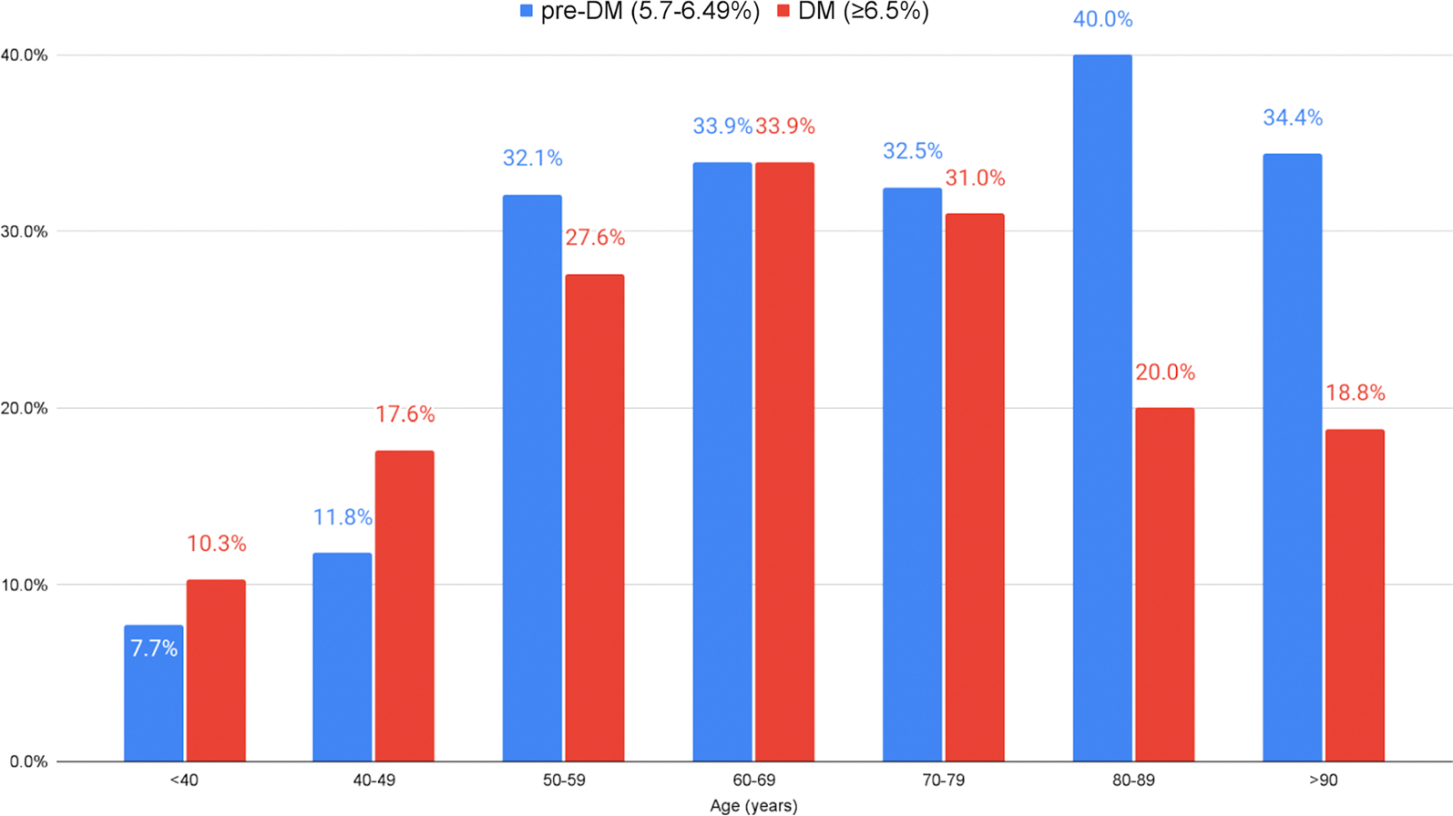
Percentage of patients in the diabetes mellitus (DM) groups stratified by age

Patients in the no-DM group were younger as compared with patients in the other groups [mean age 63.6 (95% CI 62.1–65.1) vs. 70.3 (95% CI 69.1–71.6) in the pre-DM group and 68.6 (95% CI 67.5–69.8) in the DM group; p < 0.001]. The percentage of patients in the pre-DM group increased with patients' age, while for the DM group, it peaks in the seventh decade of life and was then declines (Fig. 1). A higher proportion of men was observed in the DM group as compared with the no-DM and pre-DM groups (74% vs. 66.9% and 67%, respectively; p = 0.035). Obesity rates were higher in the pre-DM (34.1%) and DM (34.9%) groups vs. the no-DM group (24.5%) (p < 0.001). Lastly, patients in the pre-DM and DM groups had higher rates of hypertension, hyperlipidemia, chronic kidney disease, coronary artery disease, heart failure and cardiomyopathy, pulmonary hypertension, atrial fibrillation/flutter, cerebrovascular disease and peripheral artery disease as seen in Table 1.

### In-hospital complications and mortality rates

Iֹn-hospital complications rates were similar between the groups (32.6% in the no-DM group vs. 28.0%, and 26.9% in the pre-DM and DM group, respectively; p = 0.313) (Table 2). Crude in-hospital mortality rates were higher in the pre-DM and DM groups as compared with the no-DM group (3.7% and 3.0% vs. 1.5%, respectively; p = 0.072). The combined group of pre-DM and DM patients had a higher crude mortality rate when compared with no-DM patients (12.6% versus 8.2%; p = 0.01).

**Table 2:** In-hospital complications

	Groups			p-value
	HbA1c < 5.7 g% (No DM) (%)	5.7 g% ≤ HbA1c < 6.4 g% (Pre-DM) (%)	HbA1c ≥ 6.5 g% (DM) (%)	
Heart failure	4.0	3.5	5.0	0.553
Left ventricular thrombus	0.2	1.1	0.7	0.185
Shock	5.1	5.0	5.0	0.995
Recurrent MI/stent thrombosis	0.5	0.2	0.7	0.538
Malignant arrhythmia	2.7	1.7	2.2	0.580
Mechanical complication	0.2	0.4	0.5	0.678
Acute renal failure	3.1	3.5	5.0	0.308
Severe bleeding	6.2	3.5	3.7	0.077
Blood transfusions	4.1	3.9	3.1	0.862
Vascular complication	1.8	1.0	0.5	0.443
Cerebrovascular accident/transient ischemic event	1.3	0.9	1.2	0.814
Sepsis	1.8	2.0	1.2	0.685
Anoxic brain damage	0.5	0.5	0.0	0.635
Any complication	32.6	28.0	26.9	0.313
Mortality	1.5	3.7	3.0	0.072

Data are reported as n (%)

DM, diabetes mellitus; MI, myocardial infarction

### Follow-up mortality rate

**Fig. 2:**
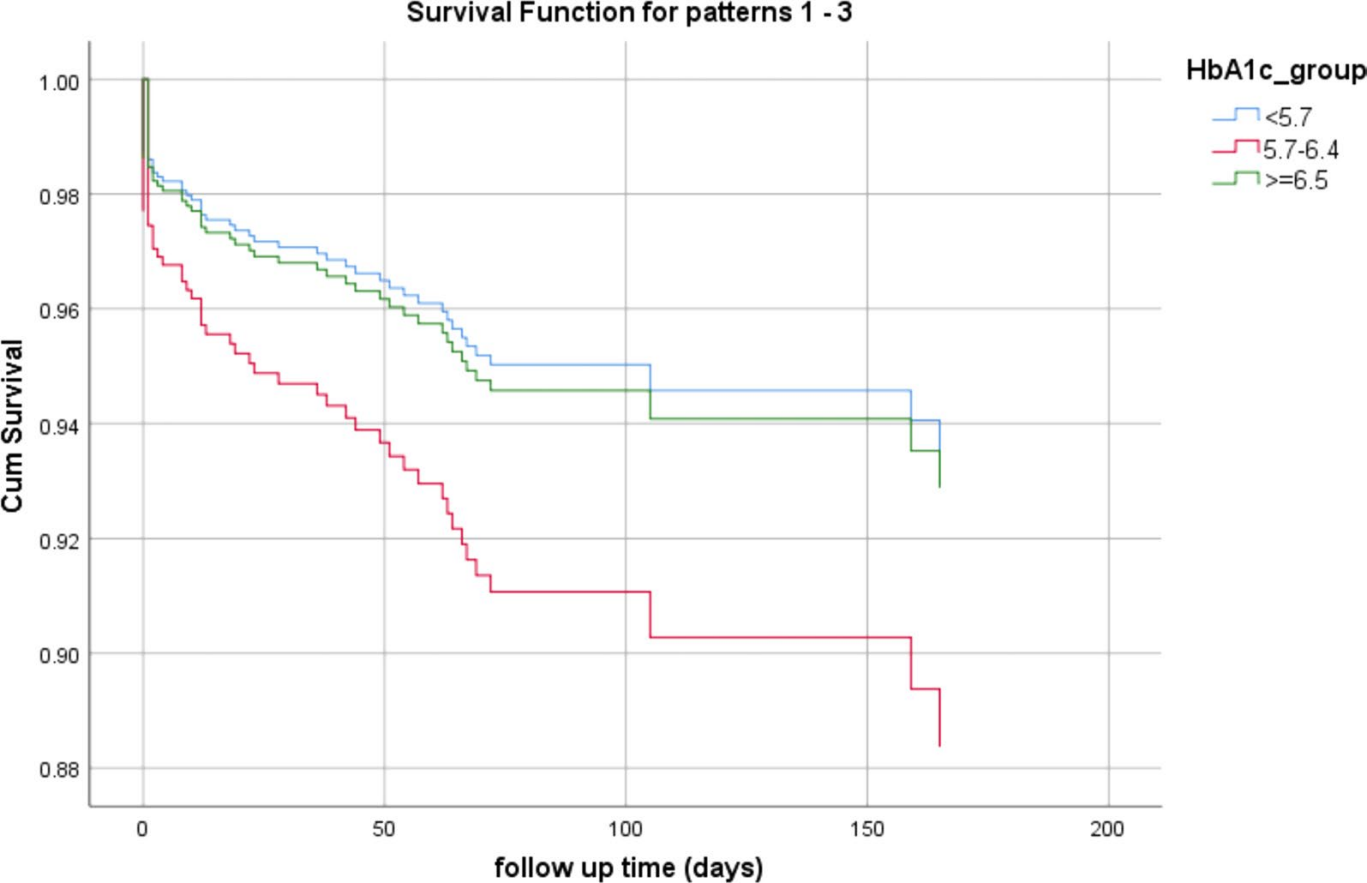
Kaplan–Meier plot for mortality by HbA1c group assignment

**Fig. 3:**
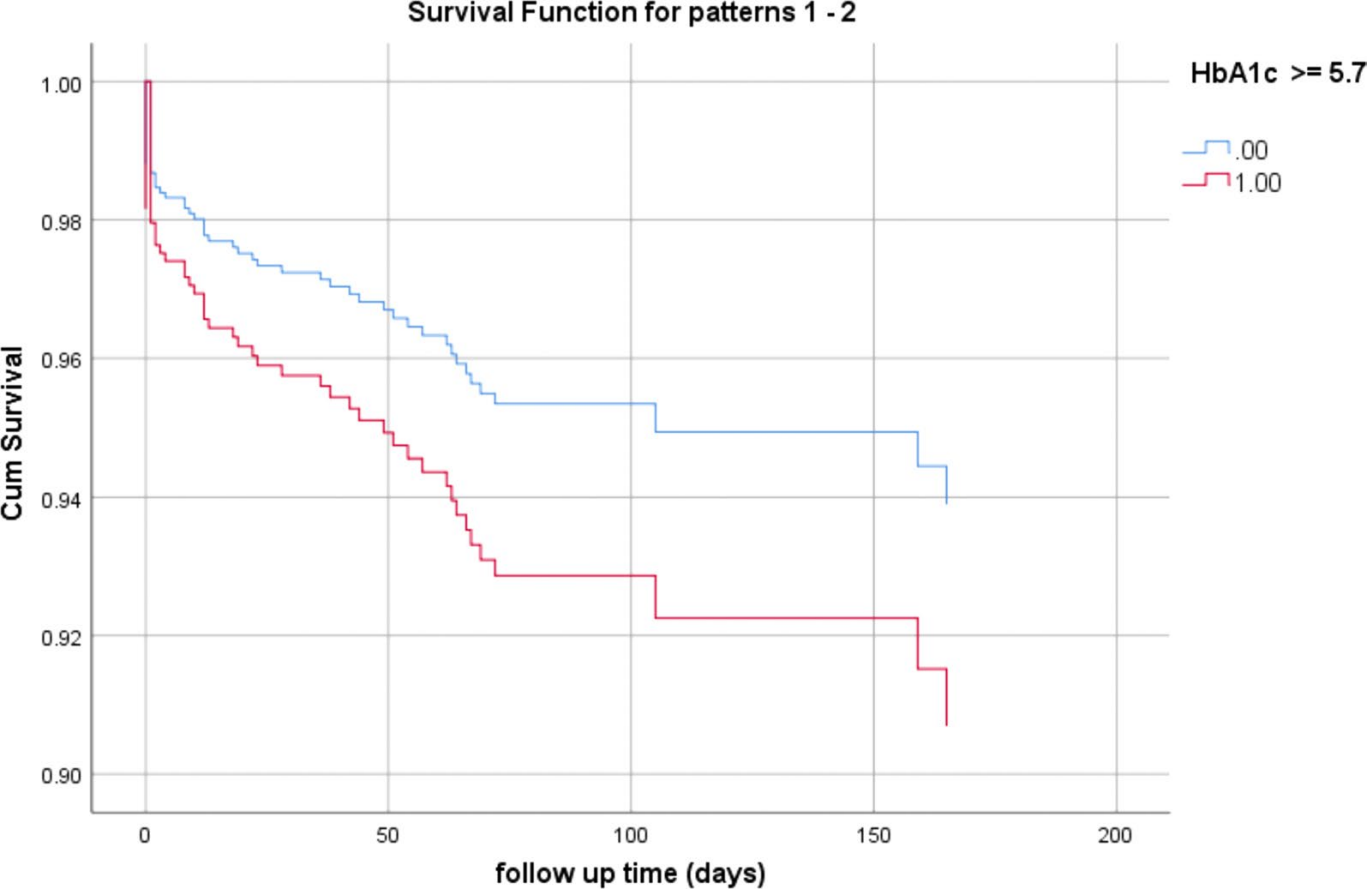
Kaplan–Meier plot for mortality by HbA1c group assignment. Pre-DM and DM are grouped together. DM, diabetes mellitus

The mortality rates during the follow-up period were 8.2% in the no-DM group vs. 12.5% and 12.8% in the pre-DM and DM group, respectively (p = 0.035). Interestingly, although in a multivariate analysis, pre-DM and DM states were associated with higher mortality rates [HR 1.84, (95% CI 0.81–2.97); p = 0.184], the Pre-DM patients had the strongest association with mortality rate [HR 1.83, (95% CI 0.936–3.588); p = 0.077]. (Tables 3 and 4; Figs. 2 and 3). Other factors found to be independently associated with mortality were: age; a history of heart failure; structural heart disease and valvular disease, and in-hospital complications (Table 3).

**Table 3:** The association between HbA1c group and mortality in a multivariate Cox logistic regression model

	HR	95% CI	p-value
Gender (male)	1.424	0.820–2.474	0.210
Age	1.059	1.030–1.088	< 0.001
Hypertension	0.968	0.492–1.906	0.926
Hyperlipidemia	1.292	0.718–2.326	0.393
A DM history	1.260	0.672–2.364	0.471
Any in-hospital complication	2.108	1.196–3.716	0.010
Prior intervention	0.450	0.239–0.846	0.013
Heart failure/cardiomyopathy/valvular heart disease	2.357	1.350–4.115	0.003
HbA1c group			
Pre-DM (5.7–6.4 g%)	1.833	0.936–3.588	0.077
DM (≥ 6.5 g%)	1.093	0.488–2.450	0.829

HR, hazard ratio; DM, diabetes mellitus

**Table 4:** The association between HbA1c group and mortality in a multivariate Cox-logistic regression model

	HR	95% CI	p-value
Gender (male)	1.438	0.827–2.501	0.198
Age	1.063	1.035–1.092	< 0.001
Hypertension	0.959	0.490–1.877	0.903
Hyperlipidemia	1.353	0.757–2.417	0.307
A DM history	1.082	0.593–1.976	0.797
Any in-hospital complication	2.206	1.257–3.872	0.006
Prior intervention	0.440	0.234–0.827	0.011
Heart failure/cardiomyopathy/valvular heart disease	2.336	1.338–4.079	0.003
HbA1c ≥ 6.5%	1.552	0.812–2.967	0.184

Pre-DM and DM are grouped together

HR, hazard ratio; DM, diabetes mellitus

## Discussion

In this large prospective trial in a tertiary care medical center ICCU, the prevalence of pre-DM (32.4%) and DM (28.6%) patients were similar to several other studies involving cardiac and ICCU patients [14, 15]. Around 9% of patients with no previous diagnosis of DM had HbA1c levels on admission in the diabetic range. Our data show that in non-selected consecutive ICCU patients the highest risk for in-hospital and overall mortality rate is among the pre-DM and DM subgroups. Surprisingly, we found that the highest mortality risk tends to be among patients with pre-DM and not in the patients with DM.

Pre-DM is an intermediate stage of glycemic control with glycemic parameters above normal but below the diabetes threshold. It is a state with a high risk of conversion to overt DM (5–10% per year) and is associated with various complications of DM, including cardiovascular complications [16]. A recent meta-analysis including more than 10-million individuals has shown that a pre-DM state is associated with an increased risk of all-cause mortality and cardiovascular disease in the general population and in patients with atherosclerotic cardiovascular disease [17]**.** However, despite the association between pre-DM and adverse cardiovascular outcomes, the recommended treatment remains focused on changing lifestyles and only suggests considering pharmacotherapy with metformin. This is in contrast to the given medical treatment and growing use of newer anti-glycemic treatments, such as sodium-glucose co-transporter-2 (SGLT2) inhibitors and glucagon-like peptide-1 (GLP1) agonists in patients with overt DM.

The pre-DM paradox, in which the highest mortality risk tends to be among patients with pre-DM, is consistent with data presented in a number of recent studies. Yahyavi et al. showed that among ambulatory patients, the 12-month risk of major adverse cardiovascular events was highest in subjects with HbA1c just below the diagnostic threshold for diabetes—the pre-DM patients [18]. They found an adjusted hazard ratio of 2.53 in the pre-DM group as compared with 2.46 in the DM group. Importantly, they also found a lower cumulative incidence of initiation of cardioprotective and glucose-lowering medications among patients just below the diagnostic threshold for DM, as compared with patients with a DM diagnosis. This might explain why those patients are at increased risk for mortality. They are less likely to receive self-management and lifestyle modification education compared with DM patients. These findings will mainly affect the long-term prognosis, as in our study. However, several other trials have shown similar findings with regard to the short-term prognosis, among a diverse group of patients. Kim et al. have found that pre-DM condition, unlike DM, was a significant predictor of short-term neurological outcomes and in-hospital mortality among patients with acute ischemic stroke [15]**.** It should be mentioned that in this study, the initial glucose measurement was higher in the DM group as compared with the pre-DM and normoglycemic groups. Hence, the higher complication rate cannot be attributed to stress hyperglycemia as demonstrated in previous studies [12]. This study also shows that pre-DM patients had a significantly lower rate of preadmission statin treatment (19.3% vs. 30%) and also lower rates of antiplatelet treatment. This, again, suggests that pre-DM patients may have been alienated from appropriate medical measures despite their cardiovascular risk. Furthermore, a recently published study investigated the impact of pre-DM and DM on the 3-year outcome of patients treated with new-generation drug-eluting stents using post-hoc analyses of two large-scale randomized clinical trials (the BIO-RESORT and BIONYX trials). This study has shown that after treatment with new-generation drug-eluting stents, both patient groups had higher risks of ischemic and bleeding events compared with non-DM patients. Differences in major bleeding were mainly attributable to dissimilarities in baseline characteristics [19]**.**

Our findings further support the findings of these previous studies that a pre-DM state is a significant risk factor for cardiovascular complications and mortality. Moreover, our study population were patients admitted to a tertiary care medical center ICCU with an acute cardiovascular disease, hence, had a higher risk for cardiovascular complications and mortality during follow-up. In light of the findings, the need arises for research regarding the effects of current diabetes treatments in patients with pre-DM state and whether the treatment recommendations for primary and secondary prevention should be changed accordingly.

### Study limitations

Our study has several limitations: First, the study was an observational study and, as such, is subjected to confounding factors. Second, it is a single-center study. Lastly, we did not have data comparing medications on admission to the different groups in the study. However, this fact is less likely to affect the external validity of the study as long as pre-DM treatment is not significantly different from what is common elsewhere. Nevertheless, our study includes a large sample size of consecutive non-selected ICCU patients and includes real-world data about patients with various cardiac diseases who required hospitalization in the ICCU, which contributes to the external validity of the study.

## Conclusion

Pre-DM and DM are common among ICCU patients. Among these patients, an HbA1c level of ≥ 5.7 g% (pre-DM & DM) is associated with a worse prognosis. Moreover, pre-DM patients probably have the highest risk for mortality following admission to the ICCU. Further studies are needed to better understand the reasons for this pre-DM paradox.

## Data Availability

The data that support the findings of this study are available from the authors [EA] upon reasonable request.

## Abbreviations

HbA1c: Hemoglobin A1C
ICCU: Intensive cardiac care unit
DM: Diabetes mellitus
ACS: Acute coronary syndrome
BMI: Body mass index
